# The engine of the reef: photobiology of the coral–algal symbiosis

**DOI:** 10.3389/fmicb.2014.00422

**Published:** 2014-08-22

**Authors:** Melissa S. Roth

**Affiliations:** Department of Plant and Microbial Biology, University of California BerkeleyBerkeley, CA, USA

**Keywords:** scleractinian corals, dinoflagellate, *Symbiodinium*, photophysiology, ecophysiology, acclimation, photoprotection

## Abstract

Coral reef ecosystems thrive in tropical oligotrophic oceans because of the relationship between corals and endosymbiotic dinoflagellate algae called *Symbiodinium*. *Symbiodinium* convert sunlight and carbon dioxide into organic carbon and oxygen to fuel coral growth and calcification, creating habitat for these diverse and productive ecosystems. Light is thus a key regulating factor shaping the productivity, physiology, and ecology of the coral holobiont. Similar to all oxygenic photoautotrophs, *Symbiodinium* must safely harvest sunlight for photosynthesis and dissipate excess energy to prevent oxidative stress. Oxidative stress is caused by environmental stressors such as those associated with global climate change, and ultimately leads to breakdown of the coral–algal symbiosis known as coral bleaching. Recently, large-scale coral bleaching events have become pervasive and frequent threatening and endangering coral reefs. Because the coral–algal symbiosis is the biological engine producing the reef, the future of coral reef ecosystems depends on the ecophysiology of the symbiosis. This review examines the photobiology of the coral–algal symbiosis with particular focus on the photophysiological responses and timescales of corals and *Symbiodinium*. Additionally, this review summarizes the light environment and its dynamics, the vulnerability of the symbiosis to oxidative stress, the abiotic and biotic factors influencing photosynthesis, the diversity of the coral–algal symbiosis, and recent advances in the field. Studies integrating physiology with the developing “omics” fields will provide new insights into the coral–algal symbiosis. Greater physiological and ecological understanding of the coral–algal symbiosis is needed for protection and conservation of coral reefs.

## INTRODUCTION

Coral reefs flourish as one of the world’s most diverse and productive ecosystems. Economic goods and ecosystem services of coral reefs are valued at over US $20 trillion annually (Costanza et al., 1997; de Groot et al., 2012). Despite their immense biological, economical, and societal significance, corals reefs are declining worldwide due to a myriad of threats on multiple scales. Synergies of global stressors (e.g., ocean warming and acidification) and local stressors (e.g., over-fishing and coastal development) accelerate the degradation of coral reefs (Hughes et al., 2003; Hoegh-Guldberg et al., 2007). Because coral reefs are at risk of global decline and corals are the keystone species of the ecosystem, it is critical to understand the dynamics of coral biology that govern responses and tolerances to environmental variability and change.

**FIGURE 1:**
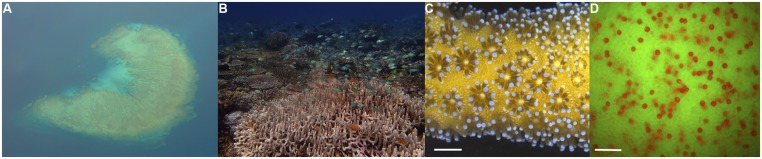
**“An oasis in a desert ocean”: coral reef seascapes powered by the coral–algal symbiosis. (A)** Aerial view of coral reef architecture in shallow, oligotrophic tropical waters of Fiji.**(B)** Reef-building corals create habitats for vibrant communities boasting incredible biodiversity and productivity. This photograph was taken in the heart of the Coral Triangle in Raja Ampat, Indonesia. **(C)** Corals are colonial invertebrates, made up of genetically identical individual polyps connected by living tissue (coenosarc). The coral golden hue of *Seriatopora hystrix* comes from symbiotic dinoflagellates located within their cells. Scale bar represents 1 cm. **(D)** The biological engine of the reef – endosymbiotic dinoflagellates of the genus *Symbiodinium* in coral cells: fluorescence microscopy image showing a *Montipora capitata* coral egg (green fluorescence from coral fluorescent proteins) and intracellular *Symbiodinium* (red fluorescence from chlorophyll). *Symbiodinium* provides photosynthetic products and oxygen to fuel coral growth and calcification. Scale bar represents 50 μm. (Images by M. S. Roth.)

Coral reefs are a paradoxical ecosystem, “an oasis in a desert ocean” (Odum, 1971), in which corals build complex structures teeming with life in shallow, oligotrophic oceans (Figures 1A,B). This calcium carbonate bioconstruction, so extensive it is visible from outer space, is powered by the coral–algal symbiosis. Dinoflagellate algae live within the cells of corals and provide their hosts with most if not all the energy needed to meet the coral’s metabolic demands (Figures 1C,D; Muscatine, 1990). Reef-building corals (phylum Cnidaria, class Anthozoa, order Scleractinia) host endosymbiotic dinoflagellates of the genus *Symbiodinium* (kingdom Chromalveolata, division Pyrrhophyta, class Dinophyceae), which are often referred to as zooxanthellae (zoo = animal and xanth = yellow) in the literature (Freudenthal, 1962). Similar to other photoautotrophs, *Symbiodinium* must delicately balance the sunlight absorbed and processed through photochemistry to sustain high rates of primary productivity without incurring damage. The fixed carbon produced by *Symbiodinium* is translocated to fuel coral growth and calcification (Goreau, 1959; Muscatine, 1990). Additionally, the oxygen produced as a by-product of photosynthesis may promote maximum coral calcification rates (Colombo-Pallotta et al., 2010). In return, corals provide their endosymbionts with essential nutrients in a safe, sunlit habitat in nutrient-poor oceans. This symbiosis is unique because it involves two eukaryotic organisms and the genome of the symbiont is three times larger than the genome of its host (Shinzato et al., 2011; Shoguchi et al., 2013). Prokaryotes and viruses are also associated with corals and *Symbiodinium*, but their roles are mostly uncharacterized (Ainsworth et al., 2010). The tight recycling and conservation of nutrients within the coral holobiont (the coral and its collective community) allows coral reefs to thrive in tropical nutrient-poor oceans. It should also be noted that there are corals without *Symbiodinium* and they do not require sunlight for nourishment nor build coral reefs and thus are not discussed in this review. The survival and success of coral reef ecosystems depend on the elegant symbiosis between reef-building corals and *Symbiodinium*.

Over the last few centuries coral reef ecosystems have endured a long trajectory of decline (Pandolfi et al., 2003), but coral reefs today face unprecedented levels of change and degradation at a global scale (Hoegh-Guldberg et al., 2007; Hoegh-Guldberg and Bruno, 2010). Changes in a suite of environmental conditions including temperature and light can lead to the breakdown and dissociation of the coral–algal symbiosis, which is called coral bleaching (Lesser, 2011). The timing and extent of coral bleaching primarily depends on the magnitude and duration of temperature anomalies as well as light levels, other environmental variables and the thermal history of the reef (Baker et al., 2008; Middlebrook et al., 2008; Strong et al., 2011). Bleached corals will die if not re-populated with *Symbiodinium*, but even recovered corals have reduced growth, regeneration, fitness, and greater susceptibility to bleaching in the future (Jokiel and Coles, 1977; Goreau and Macfarlane, 1990; Meesters and Bak, 1993; Ward et al., 2000).

Because of the central role of *Symbiodinium* photosynthesis as the engine of the coral reef ecosystem, this review summarizes the critical components and timescales of the photobiology of the coral–algal symbiosis and the underlying factors influencing the responses. This review aims to reach an audience that extends beyond photobiologists to all scientists and managers who work on coral reefs to provide them with a basic understanding of the important concepts, fundamental mechanisms and principal players in the photobiology of the coral–algal symbiosis. The extraordinary challenges confronting coral reefs require greater physiological and ecological understanding of the coral–algal symbiosis for the protection and conservation of these majestic ecosystems.

## LIGHT ENVIRONMENT OF THE CORAL–ALGAL SYMBIOSIS

Light is a key regulating factor shaping the productivity, physiology, and ecology of the coral–algal symbiosis. Light quantity (photon flux) and quality (spectral composition) are determining characteristics of the symbiosis. Both macroscale (e.g., depth) and microscale (e.g., coral skeleton structure) features influence the light environment of the symbiosis.

### LIGHT QUANTITY

**FIGURE 2:**
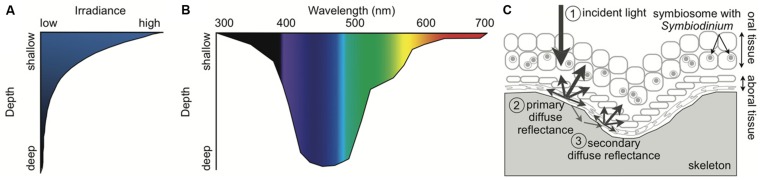
**Schematic of the light environment of the coral–algal symbiosis. (A)** Irradiance rapidly declines with depth in the ocean. **(B)** Light spectrum narrows with depth, becoming primarily blue. Wavelength attenuation properties and absorption by inorganic and organic matter in the water column determine the spectral composition at depth. **(C)** Fundamental light dynamics in the coral–algal symbiosis. (1) The principal light is downwelling incident sunlight. If the incident light is not absorbed by the coral or *Symbiodinium*, it is (2) primarily reflected by the coral skeleton as diffuse reflectance, and (3) secondarily enters the porous skeleton and then re-emerges elsewhere as diffuse reflectance. The multiple scattering by the skeleton causes the light to pass back through the coral tissue, amplifying the light *Symbiodinium* is exposed to.* Symbiodinium* is located within a host vacuole called the symbiosome (Inspired by Enríquez et al., 2005; Kirk, 2010; Marcelino et al., 2013).

To maintain high rates of productivity, coral reefs are predominantly located in shallow-waters (<30 m). In shallow-waters, corals can be exposed to high levels of downwelling irradiance of >2000 μmol photons m^-2^ s^-1^ at midday (Jimenez et al., 2012). Solar irradiance decreases exponentially with depth due to the scattering and absorbance of water itself as well as dissolved and particulate matter (**Figure 2A**; Dustan, 1982; Oliver et al., 1983; Shick et al., 1996; Lesser, 2000; Lesser et al., 2000; Kirk, 2010). Crevices, overhangs, and caves in addition to depth create low light habitats for corals. In low light environments, reef-building corals acclimate by reducing energetic requirements through decreasing tissue biomass, skeleton thickness, respiration rates, translocation, and growth (Anthony and Hoegh-Guldberg, 2003b). *Symbiodinium* in low light acclimated corals maximize the light absorption and utilization by increasing photosynthetic pigments and photosynthetic efficiency (Falkowski and Dubinsky, 1981; Anthony and Hoegh-Guldberg, 2003a,b). Reef-building corals are found throughout the photic zone with the deepest record of a reef-building coral living at 165 m (Maragos and Jokiel, 1986). Deep coral communities (>30 m), also called mesophotic coral reef ecosystems, inhabit low light environments with roughly <10% of surface irradiance (Lesser et al., 2009). Some corals such as *Montastraea cavernosa* can be found over a considerable depth range from 3 to 91 m and show a decline in gross photosynthesis and an increase in heterotrophy with depth (Lesser et al., 2010). In contrast, other corals such as *Leptoseris hawaiiensis* are restricted to the deeper zones (>60 m; Luck et al., 2013). Because of the inaccessibility of the mesophotic zone, coral physiology at these deeper depths is understudied but may provide unique insight into the coral–algal symbiosis. As sunlight penetrates seawater, the amount of direct light rapidly decreases while the amount of light from the side (diffuse light) can remain fairly constant from 10 to 40 m (Frade et al., 2008). Therefore, deeper corals experience a more uniform light field than shallower corals as well as substantially lower irradiance. At depth, in addition to the reduced irradiance, there is a narrowing of the spectrum of light present (Dustan, 1982; Shick et al., 1996; Lesser, 2000; Lesser et al., 2000; Kirk, 2010). Thus, corals from different depths not only acclimate to different light quantities, but also to distinct light quality.

### LIGHT QUALITY

The spectral composition of light changes with depth because different wavelengths have distinct attenuation properties and organic matter preferentially absorbs particular wavelengths of light (**Figure 2B**; Dustan, 1982; Shick et al., 1996; Lesser, 2000; Lesser et al., 2000; Kirk, 2010). Blue light (400–500 nm) transmits the deepest in the oceans while ultraviolet radiation (UVR, 200–400 nm) and red light (620–740 nm) attenuate the fastest (Dustan, 1982; Shick et al., 1996; Lesser, 2000; Lesser et al., 2000; Kirk, 2010). While the oceans in the tropics are oligotrophic and thus relatively transparent, reefs near coastal areas can have high amounts of dissolved organic matter (DOM) from terrigenous inputs and upwelling (Shick et al., 1996; Lesser, 2000; Zepp et al., 2008; Banaszak and Lesser, 2009; Kirk, 2010; Kuwahara et al., 2010). The absorption and scattering of DOM and in particular colored DOM (CDOM) create the unique spectral composition found on coral reefs (Shick et al., 1996; Lesser, 2000; Zepp et al., 2008; Banaszak and Lesser, 2009; Kirk, 2010; Kuwahara et al., 2010). Thus, shallow corals experience high intensity UVR and full-spectrum light (400–700 nm), while mesophotic corals experience low levels of spectrally enriched blue light.

The spectral composition of light influences corals and their symbionts on molecular, cellular, biochemical, and behavioral levels. In clear tropical oceans, high energy UVR can penetrate to >20 m and is particularly damaging for cells (Shick and Dunlap, 2002); high doses of UVR irrespective of temperature induces coral bleaching (Gleason and Wellington, 1993). While UVR can be very damaging for the coral–algal symbiosis, blue light has the greatest influence on biology and physiology. Coral photoreceptors and circadian-clock genes respond to blue light (Gorbunov and Falkowski, 2002; Levy et al., 2007). Additionally, blue light affects coral bleaching during thermal stress (Fitt and Warner, 1995), antioxidant activity (Levy et al., 2006b), coral growth and chlorophyll *a* concentrations (Kinzie et al., 1984), fluorescent protein (FP) regulation (D’Angelo et al., 2008), polyp behavior (Gorbunov and Falkowski, 2002; Levy et al., 2003), and coral regeneration (Kaniewska et al., 2009). In cyanobacteria, blue light in addition to UVR damages the photosynthetic apparatus directly and inhibits its repair (Nishiyama et al., 2006); however, whether this remains true in *Symbiodinium* is unknown. Because different pigments absorb distinct wavelengths of light, the spectral composition of light influences photosynthesis. Corals collected from 3 m have double the rates of photosynthesis under full-spectrum light as compared to blue light, while the same species collected from 40 m has double the rates of photosynthesis under blue light as compared to full-spectrum light (Mass et al., 2010b). A recent study comparing blue, red, and combined blue and red light suggests that red light alone or in combination with blue light has negative effects on symbiont health and survival (Wijgerde et al., 2014); because wavelengths of red light are attenuated quickly, only shallow corals will encounter red light. Corals and *Symbiodinium* have adapted to a variety of light environments, and light quality and quantity have significant impacts on the physiology, ecology and evolution of the photosynthetic system and the coral–algal symbiosis.

### LOCAL LIGHT ENVIRONMENT CREATED BY THE CORAL

Whereas the characteristics of the underwater light field are universal for all marine organisms within a specific location, many properties of the coral itself create a distinctive local light environment for the coral–algal symbiosis. Every component of the coral–algal symbiosis from the mucus layer to the calcium carbonate skeleton can influence the light propagating through corals and reaching *Symbiodinium* (Wangpraseurt et al., 2014). Light can be scattered, absorbed, or re-emitted as fluorescence by various components of corals and *Symbiodinium* (Kühl et al., 1995; Salih et al., 2000; Enríquez et al., 2005; Kaniewska et al., 2011; Wangpraseurt et al., 2012, 2014; Marcelino et al., 2013). The extensive genetic and environmental variability influencing each of these characteristics adds complexity to understanding the photobiology of the coral–algal symbiosis. The coral produces a highly refractive extracellular skeleton that enhances light and increases absorption (Enríquez et al., 2005). The microstructure of the skeleton creates multiple scattering of light resulting in 3–20 times higher light levels within a coral cell than in the adjacent water column (**Figure 2C**; Kühl et al., 1995; Enríquez et al., 2005; Marcelino et al., 2013). Therefore, if photons are not absorbed by the coral or its symbiont as incident light, the skeleton scatters the light as diffuse reflectance and presents more opportunities for photons to be absorbed. A recent study provides evidence that light can travel laterally a distance of ∼2 cm within the tissues of corals (Wangpraseurt et al., 2014). The light propagation properties in intact corals reduce the effects of self-shading and allow *Symbiodinium* to maximize light absorption with low investment in pigments (Enríquez et al., 2005; Wangpraseurt et al., 2012, 2014; Marcelino et al., 2013). *Symbiodinium* in corals can have high gross photosynthetic rates and quantum efficiencies can reach near theoretical limits under moderate irradiances (Rodríguez-Román et al., 2006; Brodersen et al., 2014). Early studies vary widely in reported quantum efficiencies (Dubinsky et al., 1984; Wyman et al., 1987; Lesser et al., 2000), which may have been caused by an underestimation of the absorption cross-section of chlorophyll, differences in light levels during measurements, or differences among corals in light scattering, tissue thickness, and skeletal morphology (for more discussion see Section “Photosynthesis”). Corals with complex morphologies and thick tissue encompass a variety of light microniches. Examples of light heterogeneity within a coral colony include the gradient of light through thick coral tissue and the precise location within a coral colony (e.g., the top will receive significantly more light than the bottom of a branch or the side of a colony; Kaniewska et al., 2011; Wangpraseurt et al., 2012; Brodersen et al., 2014). The light environment can determine the corals’ capacity for growth and reproduction (Goreau and Goreau, 1959; Kojis and Quinn, 1984) because corals obtain significant amounts of energy and oxygen from *Symbiodinium* primary production (Muscatine, 1990; Colombo-Pallotta et al., 2010).

## DYNAMICS OF LIGHT OF THE CORAL–ALGAL SYMBIOSIS

Light is one of the most predictable yet stochastic environmental variables of the coral–algal symbiosis. Light in the ocean is incredibly dynamic over a variety of timescales from milliseconds to thousands of years (**Table 1**). The most pronounced but consistent light cycle is the diurnal light cycle, in which *Symbiodinium* switches from producing oxygen via photosynthesis to consuming oxygen via respiration. This switch causes the environment within coral cells to change from hyperoxic during the day to hypoxic during the night (Kühl et al., 1995), and was first observed within the tissues of symbiotic sea anemones (Dykens and Shick, 1982). The amount of oxygen generated by *Symbiodinium* within coral cells can be so extensive that some corals release bubbles with high amounts of oxygen and even change the level of oxygen in the surrounding environment (D’Aoust et al., 1976; Crossland and Barnes, 1977). Coral calcification is called light-enhanced calcification because it is tightly linked with photosynthesis and corresponds with the diurnal cycle (Gattuso et al., 1999). Recent evidence suggests that the oxygen produced from photosynthesis during the day is required for maximum rates of calcification (Colombo-Pallotta et al., 2010). For more information on coral growth and calcification see reviews dedicated to the subject (e.g., Gattuso et al., 1999; Allemand et al., 2011; Tambutté et al., 2011). The diurnal light cycle and seasonal periodicity are responsible for the rhythmic responses of the circadian clock in the coral–algal symbiosis (Levy et al., 2011; Sorek et al., 2014).

**Table 1:** Timescales of light dynamics and responses by the coral–algal symbiosis.

Timescale	Light dynamics	Coral responses	*Symbiodinium* responses	Reference
<Second	Waves focus/defocuslight (e.g., wind)			L: Stramski and Dera, 1988; Falkowski and Chen, 2003; Veal et al., 2010
Seconds	Shade from floatingdebris, swimminganimals, etc.			
Minutes	Clouds	Polyp tentacle contraction	qE qT Xanthophyll cycling Functional absorptioncross-section of PSII Enzyme activity regulation* Protein degradation*	L: Falkowski and Chen, 2003; C: Levy et al., 2003; *S*: Brown et al., 1999; Jones and Hoegh-Guldberg, 2001; Hill and Ralph, 2005; Ralph et al., 2005; Levy et al., 2006a; Eberhard et al., 2008; Hill et al., 2012
Hours	Tide (daily low to high) Diurnal cycle	Extreme tissue retraction (off parts of skeleton) Calcification Gene expression of cryptochromes, antioxidants, and carbonic anhydrase Antioxidant activity Diurnal tentacle expansion/contraction	qI Δ*F*/*F*_m_^′^ D1 repair Photosynthetic pigments Antioxidant activity Oxygen-evolving enhancer 1expression (*OEE1*) Transcriptional changes	L: Brown et al., 1994; Falkowski and Chen, 2003; Jimenez et al., 2012; C: Kawaguti, 1954; Brown et al., 1994; Gattuso et al., 1999; Levy et al., 2006a, 2011; *S*: Warner et al., 1999; Gorbunov et al., 2001; Levy et al., 2006a; Jimenez et al., 2012; Sorek et al., 2013, 2014
Days	Vertical mixing Clouds Storms	Antioxidant activity MAAs	*F*_v_/*F*_m_ Antioxidant activity MAAs	L: Falkowski and Chen, 2003; Anthony et al., 2004; C: Shick, 2004; Levy et al., 2006b; Shinzato et al., 2011; *S*: Shick and Dunlap, 2002; Shick, 2004; Levy et al., 2006b; Roth et al., 2010
Weeks	Tide (sun and moon alignment) Turbidity Clouds	FP gene expression GFP concentration Growth rate	Density Chlorophyll *a* and *c_2_* Peridinin Xanthophyll pool β-carotene rETR Saturating irradiance	L: Anthony et al., 2004; C: Bay et al., 2009; Roth et al., 2010; *S*: Falkowski and Dubinsky, 1981; Anthony and Hoegh-Guldberg, 2003a; Bay et al., 2009; Roth et al., 2010
Months		FP concentration	Xanthophylls Light-harvesting complexes PSI reaction center Number of photosynthetic units Optical cross-section of the photosynthetic unit *P*_max_ Respiration	C: Dove et al., 2008; *S*: Iglesias-Prieto and Trench, 1994, 1997; Anthony and Hoegh-Guldberg, 2003a; Dove et al., 2008
Seasons	Season (e.g., day length, solar declination cycle)	Tissue biomass	Chlorophyll *a* Chlorophyll *a*:*c_2_* Δ*F*/*F*_m_^′^ *F*_v_/*F*_m_ Density Phylotype	L: Kirk, 2010, C: Fitt et al., 2000, *S* Fagoonee et al., 1999; Fitt et al., 2000; Warner et al., 2002; Suwa et al., 2008; Ulstrup et al., 2008
∼Year		Gross skeleton morphology		C: Muko et al., 2000
≫Years	Ozone depletion Global dimming/brightening Orbital selection	Adaptation Speciation	Adaptation Speciation	L: Falkowski and Chen, 2003; Banaszak and Lesser, 2009

References for light dynamics, coral responses, and *Symbiodinium* responses classified as “L,” “C,” and “*S*,” respectively.

*General timescale for photosynthetic eukaryotes.

During the day, many factors influence the amount of solar energy the coral–algal symbiosis receives. Waves on the surface of the ocean act as lenses causing the sunlight to focus and defocus creating 100-fold changes in light intensity on millisecond timescales (Stramski and Dera, 1988; Falkowski and Chen, 2003). Sunlight flashes in shallow-waters can exceed 9000 μmol photons m^-2^ s^-1^ and occur >350 times per minute (Veal et al., 2010). Additionally, marine organisms such as fish swim over corals and temporarily shade them. Shading from clouds and storms can reduce irradiance by 40-fold and last for minutes or weeks (Falkowski and Chen, 2003; Anthony et al., 2004). The irradiance of corals is also affected by the tidal cycle, which alters the depth of the water column and can even cause shallow corals to become subaerially exposed during extreme low tides (Brown et al., 1994; Anthony et al., 2004; Jimenez et al., 2012). Throughout the year, changes in day length and solar declination modify the amount of sunlight available (Kirk, 2010). It should be noted that light is not only an indirect source of energy for corals, but also provides informational signals for reproduction and spawning, which are tightly linked to the lunar cycle (Harrison and Wallace, 1990; Levy et al., 2007). The complex dynamics of interweaving random and cyclic processes that govern light availability have profound effects on photosynthesis and coral–algal physiology.

## PHOTOSYNTHETIC SYMBIOSES IN CORALS INCREASE SUSCEPTIBILITY TO OXIDATIVE STRESS

Photosynthesis, the conversion from solar energy to chemical energy, is one of the most important processes on our planet. Using sunlight, oxygenic photosynthetic organisms, such as *Symbiodinium,* convert carbon dioxide and water into organic carbon. This process also generates oxygen, which supports aerobic life on Earth. In reef-building corals, photosynthesis by *Symbiodinium* provides most of the energy needed for corals to build the infrastructure of the reef (Goreau, 1959; Muscatine, 1990). The primary photosynthetic pigments of *Symbiodinium,* chlorophyll *a*, chlorophyll *c_2_*, and peridinin, determine which wavelengths of light are utilized in photosynthesis (**Table 2**). Light-harvesting complexes capture photons of light and transfer the energy to the photosynthetic electron transport chain. Light-induced linear electron flow from water to NADPH involves electron transfer from photosystem II (PSII) to photosystem I (PSI) via the cytochrome *b_6_f* complex to generate ATP (for diagram of arrangement see Eberhard et al., 2008). Cyclic electron flow must run in concert with linear electron transport for efficient photosynthesis (Munekage et al., 2004). Cyclic electron flow utilizes PSI and cytochrome *b_6_f* to build a high proton motive force and thus ATP. Photosynthetically derived NADPH and ATP are used to drive the fixation of carbon dioxide in the Calvin–Benson cycle as well as other metabolic processes in the chloroplast. The reaction centers, PSI and PSII, are embedded in the thylakoid membrane of the chloroplast.

**Table 2:** Summary of light absorbing and emitting pigments, proteins, and compounds in the coral--algal symbiosis.

Pigment/protein/compound	Produced by	λ_abs_ (nm)	λ_em_ (nm)	Reference
Chlorophyll *a*	*Symbiodinium*	435^a^–440^b^ (670–680^a,b^)	678^c^, (735^c^)	Govindjee and Braun, 1974; Iglesias-Prieto et al., 1993; Bricaud et al., 2004
Chlorophyll *c*_2_	*Symbiodinium*	450–460^b^	644^d^	Iglesias-Prieto et al., 1993; Bricaud et al., 2004; Johnsen et al., 2011
Peridinin	*Symbiodinium*	478–500^b^	NA	Bricaud et al., 2004; Johnsen et al., 2011
Diadinoxanthin	*Symbiodinium*	460^b^(~490^b^)	NA	Bricaud et al., 2004
Diatoxanthin	*Symbiodinium*	449^e^ (475^e^)	NA	Bricaud et al., 2004
*β-carotene*	*Symbiodinium*	460^b^ (~490^b^)	NA	Bricaud et al., 2004
CFP	Coral	404–477	483–495	Salih et al., 2000; Alieva et al., 2008; D’Angelo et al., 2008; Gruber et al., 2008; Roth et al., 2013
GFP	Coral	470–512	497–525	Salih et al., 2000; Matz et al., 2002; Mazel et al., 2003; Alieva et al., 2008; D’Angelo et al., 2008; Gruber et al., 2008; Roth et al., 2010, 2013
RFP	Coral	556–597	572–609	Salih et al., 2000; Matz et al., 2002; Mazel et al., 2003; Alieva et al., 2008; D’Angelo et al., 2008; Gruber et al., 2008; Palmer et al., 2009b
CP	Coral	560–590	NA	Dove et al., 1995; Matz et al., 2002; Alieva et al., 2008; D’Angelo et al., 2008
MAAs	Coral/*Symbiodinium*	310–360	NA	Shick and Dunlap, 2002; Shinzato et al., 2011

*Secondary peaks listed in parentheses.*

*^a^Measured in algal cells.*

*^b^Estimated in vivo absorption.*

*^c^Measured in purified thylakoid membranes.*

*^d^Measured in purified PSII.*

*^e^Measured in ethanol extracts.*

While endosymbiont photosynthesis serves as the engine to power the growth and calcification of coral reefs, sunlight capture, absorption, and utilization presents a high potential for photo-oxidative damage. Oxidative stress results from the production and accumulation of reactive oxygen species (ROS) and can damage lipids, proteins and DNA and signal cell apoptosis or exocytosis (Gates et al., 1992; Lesser, 1997; Hoegh-Guldberg, 1999; Franklin et al., 2004; Lesser and Farrell, 2004; Lesser, 2006). Oxidative stress is considered the unifying mechanism for a number of environmental insults that elicit coral bleaching (Lesser, 2011), resulting in the loss of *Symbiodinium* from host cells via mechanisms such as apoptosis, exocytosis, and necrosis (reviewed in Gates et al., 1992).

**FIGURE 3:**
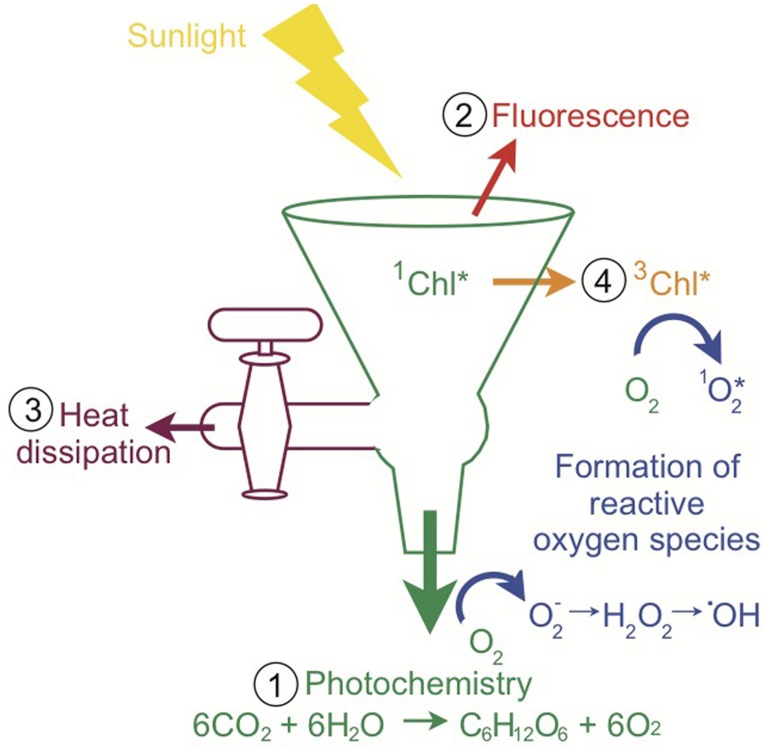
**Pathways of light energy utilization by *Symbiodinium*.** The funnel scheme of the photosynthetic apparatus depicts the possible fates of absorbed light. When sunlight is absorbed by chlorophyll, the singlet-state excitation of chlorophyll (^1^Chl*) is formed and the excitation energy can be (1) used to drive photochemistry, (2) re-emitted as fluorescence, (3) dissipated as heat (NPQ), or (4) decayed via the chlorophyll triplet state (^3^Chl*), which produces reactive oxygen species as a by-product. Multiple types of reactive oxygen species are produced during photosynthetic electron flow. When the light exceeds what can be processed through these pathways, there is a high potential for the accumulation of reactive oxygen species and ultimately oxidative stress (inspired by Müller et al., 2001 and Demmig-Adams and Adams, 2002).

Although light is required for photosynthesis, excess light can be extraordinarily harmful for photosynthetic organisms and their hosts. There are four main fates for sunlight absorbed by a photosynthetic organism, depicted in the “funnel scheme” in **Figure 3**. The principal role for absorbed sunlight is to drive the photochemical reactions of photosynthesis. However, due to the dynamic nature of sunlight, the photosynthetic apparatus often receives more light than can be processed through photochemistry and the excess light must be diverted away from carbon assimilation and utilized by other pathways to minimize photo-oxidative damage (Niyogi, 1999; Müller et al., 2001). The absorbed excitation energy can also be re-emitted as chlorophyll fluorescence (red light), dissipated as heat which is termed non-photochemical quenching (NPQ), or decayed via the chlorophyll triplet state in which ROS are produced (**Figure 3**; Asada, 1999; Müller et al., 2001). On a sunny day, *Symbiodinium* in shallow corals dissipate four times more light energy than is used in photosynthesis (Gorbunov et al., 2001). Experimentally, corals under typical irradiances of coral reefs (640 μmol photons m^-2^ s^-1^) dissipate 96% of the energy and use only 4% of absorbed light energy for photosynthesis (Brodersen et al., 2014). Highly reactive intermediates and by-products such as ROS can cause photo-oxidative damage to the photosynthetic apparatus and are inevitably produced during photosynthesis (Niyogi, 1999). Therefore, the photosynthetic system is constantly repairing itself from the damage (Niyogi, 1999). If the rate of damage exceeds the rate of repair, there will be reductions in photosynthetic efficiency and/or maximum rates of photosynthesis, which is called photoinhibition (Niyogi, 1999). Oxidative damage can decrease the outflow from the funnel, which intensifies the problem through increased production of ROS (**Figure 3**). Consequently, photosynthetic organisms have numerous photoprotective strategies. For example, adjusting the size of the light-harvesting complexes (volume of the funnel), photosynthetic capacity (rate of the primary outflow of the funnel), and NPQ capacity (rate of the secondary outflow of the funnel) can vary how much energy can be accommodated and how much excess energy or spillover there is. The rates of photochemical reactions and turnover rates of electron sinks (outflow from the funnel) are sensitive to changes in temperature and low temperatures can cause an energy imbalance and overexcitation of PSII (Huner et al., 1998; Nobel, 2005). Additionally, increases in temperature can change the repair rates of photosynthetic proteins and thus indirectly affect outflow from the funnel (Warner et al., 1999; Takahashi et al., 2004). Changes in temperature can also disturb thylakoid membrane fluidity and decrease the outflow from the funnel through the uncoupling of photosynthetic energy transduction and a reduction in carbon assimilation from the leaking of protons and consequently decrease ATP production (Tchernov et al., 2004). Other photoprotective processes include photorespiration, water–water cycle, antioxidant systems, and repair and new synthesis of proteins (Niyogi, 1999). Photosynthetic organisms balance the light entering and exiting the photosynthetic apparatus (the funnel) to maximize photosynthesis under the conditions the organism lives in while preventing oxidative damage. Excess light (flow into the funnel) and/or changes in temperature (direct and indirect effects of flow out of the funnel) are principal factors causing energy imbalance in photosynthetic organisms (Huner et al., 1998; Nobel, 2005). All of these processes ultimately influence the health of the coral–algal symbiosis and the propensity for bleaching.

The photosynthetic apparatus is a flexible molecular machine that is highly conserved among eukaryotes (Falkowski and Raven, 2007; Eberhard et al., 2008). However, *Symbiodinium* photosynthesis occurs within an animal cell creating additional complexities. During the day, *Symbiodinium* generates high amounts of oxygen as a by-product of photosynthesis. Despite the fact that the coral absorbs oxygen during respiration, the coral cell becomes hyperoxic and may even produce bubbles of oxygen (D’Aoust et al., 1976; Crossland and Barnes, 1977; Kühl et al., 1995). The excess oxygen makes both the coral and its symbiont susceptible to oxidative stress (Lesser, 2006). Because the highly reflective coral skeleton enhances light within the coral cell, the loss of photosynthetic pigments and/or symbionts through coral bleaching increases the local irradiance and aggravates the negative effects of the stressful environmental conditions (**Figure 2C**; Enríquez et al., 2005; Marcelino et al., 2013): bleaching can result in 150% increase in scalar irradiance within coral tissues as compared to a healthy coral (Wangpraseurt et al., 2012). *Symbiodinium* photosynthesis is sensitive to changes in temperature and light (Lesser et al., 1990; Iglesias-Prieto et al., 1992; Lesser and Farrell, 2004; Roth et al., 2012; Downs et al., 2013). A recent study on *Symbiodinium* in corals provides evidence that light stress without heat stress causes fusion of thylakoid lamellae concurrent with photo-oxidative damage, heat stress without light stress causes decomposition of thylakoid structures which consequently generates photo-oxidative stress, and combined heat and light stresses induce both pathomorphologies (Downs et al., 2013). In nature, heat stress that produces coral bleaching generally occurs over weeks (Strong et al., 2011), which would mean that heat stress will be concurrent with daylight. Season, cloud cover, water clarity, and waves among other parameters determine the irradiance levels corals are exposed to. Because *Symbiodinium* is generally more susceptible to heat stress than their coral hosts (Strychar and Sammarco, 2009), *Symbiodinium* can become a substantial source of ROS during heat stress (Yakovleva et al., 2009). Excess ROS is transferred to and accumulates in the host (Levy et al., 2006b), and correspondingly the gene expression response to heat stress is larger in the coral than the symbiont (Leggat et al., 2011a). When corals bleach the main source of ROS production is removed although it is important to acknowledge that the host itself may also be producing ROS (Lesser, 2006; Weis, 2008). The delicate balance of *Symbiodinium* light absorption and utilization within the hyperoxic cells of corals in a dynamic environment makes the coral–algal symbiosis vulnerable to oxidative stress.

Because of the importance of oxidative stress in the coral–algal symbiosis and its role in coral bleaching, a brief discussion of ROS production, damage, and cellular defenses is warranted. There are a variety of types of ROS, with different degrees of reactivity and diffusivity across membranes, including: singlet oxygen (^1^O_2_^∗^), superoxide (O_2_^-^), hydrogen peroxide (H_2_O_2_), hydroxyl radical (**^.^**OH), and the reactive nitrogen species nitric oxide (NO) and peroxynitrite anion (ONOO^-^; Lesser, 2006). The major sites of ROS production are the chloroplast (light-harvesting complexes, PSI and PSII), mitochondria (inner membrane), and the endoplasmic reticulum (Niyogi, 1999; Lesser, 2006). The main targets of oxidative damage in *Symbiodinium* are the D1 protein of PSII and its repair mechanism, the enzyme ribulose 1,5-bisphosphate decarboxylase/oxygenase (Rubisco) of the Calvin–Benson cycle, and thylakoid membranes (Lesser, 1996; Warner et al., 1999; Takahashi et al., 2004; Tchernov et al., 2004). The cellular mechanisms of photoinhibition and coral bleaching are not described here as the topic has been recently reviewed elsewhere (see Lesser, 2006, 2011; Weis, 2008). The coral–algal symbiosis has an arsenal of defenses to combat ROS and neutralize damage including the antioxidant enzymes superoxide dismutase (SOD), catalase (CAT), and peroxidase, and the nonenzymatic antioxidants ascorbic acid, glutathione, tocopherol, carotenoids, uric acid, dimethylsulfide, dimethylsulphoniopropionate, and mycosporine-like amino acids (MAAs; Lesser, 2006). SOD catalyzes O_2_^-^ into H_2_O_2_ and O_2_, while CAT and peroxidase catalyze the H_2_O_2_ into H_2_O and O_2_. There is some evidence that accumulation of H_2_O_2_ is the primary ROS causing loss of *Symbiodinium* in corals (Sandeman, 2006). Recently, it has been suggested that enzyme mitochondrial alternative oxidase (AOX) in *Symbiodinium* could compete for electrons and reduce oxidative stress in the mitochondria (Oakley et al., 2014). In addition to the suite of photoprotective defenses, the coral–algal symbiosis employs a variety of approaches to optimize photosynthesis to maintain high rates of productivity under distinct light environments (see Sections “Photobiology of Corals” and “Photobiology of *Symbiodinium*”).

## PHOTOBIOLOGY OF CORALS

Extreme light intensity can be directly damaging for corals as well as indirectly harmful via the cascade of events that can occur from photo-oxidative damage. Corals may control the light *Symbiodinium* receives because the symbiont is located within the host oral endoderm cells inside a vacuole called the symbiosome (**Figure 2C**). Most corals live in shallow, oligotrophic habitats characterized by high light. However, corals also inhabit low light environments such as in caves or under overhangs in the shallows or in the mesophotic zone, where light becomes diffuse and monochromatic. Because adult corals are sessile, long-term acclimation to their particular light environment results in dramatic differences between high and low light corals (Falkowski and Dubinsky, 1981; Anthony and Hoegh-Guldberg, 2003b). Corals regulate antioxidants, pigments, gene expression, behavior, and architectural levels over multiple timescales in response to changes in ambient light to optimize fitness of the coral–algal symbiosis (**Table 1**).

### ENZYMATIC ANTIOXIDANTS

Because photosynthesis invariably produces ROS, enzymes that can neutralize ROS have a fundamental role in coral photophysiology. Corals synthesize the enzymes SOD and CAT (Shick et al., 1995), which work together to convert O_2_^-^ and H_2_O_2_ into H_2_O and O_2_. The activities of SOD but not CAT decline with depth in shallow corals suggesting a relationship with the potential for oxidative stress (Shick et al., 1995). Both SOD and CAT activities have a diurnal pattern increasing with light and photosynthesis and decreasing at night (Levy et al., 2006a). In just two days, corals significantly increase the activities of SOD and CAT under blue light and decrease the activities in prolonged darkness (Levy et al., 2006a,b). Levy et al. (2006a) found that the response in antioxidants by the host was larger than the symbiont. As expected, gene expression of at least some antioxidants is coupled with the diurnal light cycle (Levy et al., 2011).

### MYCOSPORINE-LIKE AMINO ACIDS

Because mycosporine-like amino acids are small molecules that absorb UVR and have antioxidant activities (**Table 2**), they play an essential function in photoprotection of marine organisms (Shick and Dunlap, 2002). While MAAs accumulate in host tissues (Shick and Dunlap, 2002), it is unclear which partner of the symbiosis synthesizes them. Originally it was presumed that MAAs were synthesized by *Symbiodinium* because of their presence in *Symbiodinium* in culture (Shick and Dunlap, 2002); however, the recent sequencing of the coral genome *Acropora digitifera* shows that the host also has the genes required for the biosynthesis of MAAs (Shinzato et al., 2011). It is also hypothesized that corals can acquire MAAs through their diet (Shick and Dunlap, 2002). Changes in concentration of MAAs occur over days with primary MAAs appearing first and secondary MAAs, which are synthesized from precursor MAAs, developing later (Shick, 2004). MAAs are also found in the coral mucus where they absorb nearly ∼10% of UVR (Teai et al., 1998). It is currently unknown which partner contributes which types of MAAs found in the coral–algal symbiosis. Because MAAs are thermally stable, they may play an important role scavenging free radicals and quenching singlet oxygen during heat stress (Banaszak and Lesser, 2009).

### FLUORESCENT PROTEINS

Fluorescent proteins are proteins that absorb higher energy light and re-emit lower energy light. Corals produce a variety of FPs that absorb between ∼400–600 nm and fluoresce between ∼480–610 nm with Stokes shifts ranging from ∼10–90 nm (**Table 2**; Alieva et al., 2008). The most common FP is the green FP (GFP), but corals also produce cyan FPs (CFP), red FPs (RFP), and even those that only absorb light but do not fluoresce called chromoproteins (CP; **Table 2**; Salih et al., 2000; Matz et al., 2002; Alieva et al., 2008; Gruber et al., 2008). The FP superfamily exhibits diversity in color while remaining similar on a structural level (Tsien, 1998). The three-dimensional structure, an 11-stranded β-barrel fold and a central α-helix containing the three amino acid chromophore, makes *in vitro* FPs stable and resistant to changes in temperature and pH (Tsien, 1998). FPs contribute to the vivid coloration of corals (Dove et al., 2001; Oswald et al., 2007). Corals synthesize high concentrations of FPs and are ubiquitous in shallow reef-building corals (Salih et al., 2000; Leutenegger et al., 2007) as well as in mesophotic reef-building corals (Roth et al., in review).

Light regulates FP expression in corals. Corals increase and decrease GFP concentrations within 15 days in response to increased and decreased light, respectively (Roth et al., 2010). Green light and to an even greater extent blue light increases gene expression of CFP, GFP, RFP, and CP (D’Angelo et al., 2008). However, a field study did not show a significant correlation between depth and GFP concentration in *M. cavernosa* and* M. faveolata* (Mazel et al., 2003). Coral larvae and adults of the same species, which are found in different light environments, can express distinct FPs (Roth et al., 2013). Additionally in the mesophotic zone (>60 m), the type of FP is correlated with depth to match the spectral quality of light both within species as well as among closely related species (Roth et al., in review).

The function of FPs remains ambiguous and controversial despite being prevalent on coral reefs as well as within corals where they make up a significant portion of the total soluble protein (Salih et al., 2000; Leutenegger et al., 2007; Roth et al., in review). The high diversity of both corals and FPs may create challenges to understanding the functions because different FPs could have unique roles in different species. The predominant hypotheses on the functions of FPs include photoprotection (either directly by absorbing harmful light energy or indirectly as an antioxidant) and photosynthesis enhancement (Kawaguti, 1944, 1969; Salih et al., 2000; Bou-Abdallah et al., 2006; Palmer et al., 2009a). Some corals express multiple types of FPs and the emission spectra of some FPs overlap with the absorption spectra of other FPs providing the possibility for higher energy to be reduced to lower energy via fluorescence resonance energy transfer between FPs within corals (**Table 2**; Salih et al., 2000). Despite the tight relationship between light and FPs (Vermeij et al., 2002; D’Angelo et al., 2008; Roth et al., 2010), evidence against a photoprotective hypothesis includes a lack of correlation between depth and GFP as well as the negligible impact of GFP absorption, emission, and reflection on sunlight reaching *Symbiodinium* (Mazel et al., 2003). However, recent evidence suggests that CPs can reduce chlorophyll excitation and thus may serve a direct photoprotective role (Smith et al., 2013). Moreover, FPs decrease susceptibility to coral bleaching during heat stress providing more evidence for a photoprotective role (Salih et al., 2000). CP concentration is strongly correlated with photosynthetic capacity at the onset of bleaching (Dove et al., 2008), which may suggest that FPs plan an important role in mitigating thermal stress for the symbiont. FPs have also been shown to have antioxidant activity, which could provide an indirect photoprotective role (Bou-Abdallah et al., 2006; Palmer et al., 2009a). This activity may explain why under temperature stress GFP is rapidly degraded or used up (Roth and Deheyn, 2013). In contrast, there is much less supporting evidence for the photosynthesis enhancement hypothesis. The emission of FPs and the absorption of photosynthetic pigments are not aligned (**Table 2**); there is inefficient energy transfer between host and *Symbiodinium* pigments (Gilmore et al., 2003) and GFP emission has negligible impact on light reaching *Symbiodinium* (Mazel et al., 2003). Additionally, there are no differences in abundance, photophysiology, or genotype of *Symbiodinium* in mesophotic corals with and without coral fluorescence (Roth et al., in review). Nevertheless, the high abundance of fluorescence in energetically limited corals of the mesophotic zone suggests that FPs play an integral physiological role (Roth et al., in review).

The visual nature of FPs and the strong correlation with growth enables coral fluorescence to be utilized as an indicator of coral health (Roth et al., 2010; D’Angelo et al., 2012; Roth and Deheyn, 2013). During temperature stress, there is a rapid decline in GFP prior to coral bleaching providing an early signal of declining coral condition (Roth and Deheyn, 2013). While the function of FPs is uncertain, it is clear they are involved in the photophysiological response of the coral–algal symbiosis.

### TISSUE THICKNESS

Tissue thickness directly affects the amount of light reaching *Symbiodinium.* Photosynthetically active radiation (PAR, 400–700 nm) decreases within the coral tissue while near-infrared radiation (NIR, 700–800 nm) is consistent throughout the coral tissue (Wangpraseurt et al., 2012). In Caribbean corals, the tissue thickness is highest in the spring and the lowest in the summer-fall when there are lower energetic reserves, which also correlates with changes in *Symbiodinium* density (Fitt et al., 2000). It is hypothesized that an increase in translocated photosynthetic products associated with proliferating *Symbiodinium* density must precede the enlargement in tissue biomass (Fitt et al., 2000). Small changes in the tissue thickness will affect the amount of light penetrating the coral as well as the amount of multiple scattering.

### POLYP BEHAVIOR

Despite living as a sessile organism, corals have adapted a unique set of behaviors to regulate light exposure. Coral polyp size varies greatly, from less than 1 cm (**Figure 1C**) to greater than 30 cm in length in solitary corals (e.g., *Fungia*). Polyp size affects the surface area to volume ratio and in most corals is inversely related to photosynthesis and respiration (Porter, 1976). The polyp behavior, extension and contraction, can dramatically affect the light environment within coral cells. Corals can retract their polyps in minutes in response to high light (Levy et al., 2003) and as part of the diurnal cycle (Kawaguti, 1954). For heterotrophic feeding, corals extend their polyps to capture prey, but this primarily occurs at night. Intertidal corals can become exposed to high light and air during extreme low tides and have developed unique adaptations including the reversible retraction of coral tissue deep into the skeleton so that the tissue is no longer visible (Brown et al., 1994). During the extreme tissue retraction, the white bare coral skeleton increases the albedo and reduces the sunlight absorbed. Furthermore, the pigments in the tissue are condensed and the amount of light is decreased within coral cells. The tips of the tentacles are often distinctly pigmented (**Figure 1C**) and it has been suggested that FPs can act as a sunscreen plug when the polyp is retracted (Salih et al., 2000). *Symbiodinium* are located in coral cells both in the polyp and the coenosarc (tissue that connects polyps), however only the polyp can be extended or retracted. An extended polyp increases the surface area to volume ratio allowing for faster diffusion of carbon dioxide and oxygen in and out of coral cells. Additionally, a greater amount of lateral light is transmitted when the polyp is extended (Wangpraseurt et al., 2014). The surface irradiance over polyps is higher than over the coenosarc (Wangpraseurt et al., 2012). The differences in light and/or photosynthetic substrates may be responsible for the spatial heterogeneity observed in photosynthetic responses (Ralph et al., 2002).

### SKELETON MORPHOLOGY

Scleractinian corals have tremendous phenotypic plasticity in morphology. Light, in addition to water flow, is one of the primary influences on morphology (Todd, 2008). Gross morphology determines the exposure of the coral–algal symbiosis to different light regimes, while microscale morphology and skeleton composition can influence light scattering. Even within a species, corals become flatter under low light to enhance light capture and more branched under high light to augment self-shading (Muko et al., 2000; Padilla-Gamiño et al., 2012); changes in gross morphology can occur in less than a year (Muko et al., 2000). Depending on morphology, the top, sides, and bottom of a coral can have dramatically different light environments (Warner and Berry-Lowe, 2006; Kaniewska et al., 2011). In chronic low light environments such as caves, overhangs and at depth, corals have a plate-like flat morphology and thinner skeleton (Kühlmann, 1983; Anthony and Hoegh-Guldberg, 2003b). Because multiple scattering by the coral skeleton amplifies light within the coral cells (Enríquez et al., 2005), the microscale architecture dictates the light field the symbionts are exposed to. Light within coral cells can differ dramatically depending on the precise location of the tissue; for example, there is higher irradiance in cells on top of ridges than in cells between ridges (Kühl et al., 1995). Corals have diverse skeletal fractality on nano- and microscales that causes an eightfold variation in the light scattering properties (Marcelino et al., 2013). Lastly, corals can vary how much of their tissue penetrates the skeleton. Corals that are perforate, porous skeletal matrices with intercalating tissue, can have five times thicker tissues than imperforate corals, those with tissue that do not penetrate the skeleton (Yost et al., 2013). Light and coral morphology are intricately interconnected and morphology creates conspicuous light microenvironments.

From small molecules and proteins to behavior and morphology, corals employ many strategies to modify the light environment within the coral cell. While the various strategies to alter light are known, many of the molecular, cellular, and biochemical processes to regulate these methods are understudied. In contrast, the cellular and biochemical photophysiology of *Symbiodinium* is much better understood.

## PHOTOBIOLOGY OF *Symbiodinium*

Corals are highly refractive and provide an environment where *Symbiodinium* have high gross rates of photosynthesis and quantum efficiencies close to their theoretical limits (Rodríguez-Román et al., 2006; Brodersen et al., 2014). Because light is the driving force of photosynthesis, photophysiology of photosynthetic organisms has been a very active area of research. *Symbiodinium* optimizes the amount of light absorbed and utilized by photochemistry, while shunting light when the photosynthetic capacity has been reached. On sunny days, ∼80% of light is dissipated by *Symbiodinium* in shallow corals and not used in photochemistry (Gorbunov et al., 2001). Experimental measurements confirm that corals dissipate 96% of absorbed light energy under typical irradiances of coral reefs (640 μmol photons m^-2^ s^-1^; Brodersen et al., 2014). Sunlight flashes dramatically increase light in milliseconds, but have little effect on overall photosynthesis of *Symbiodinium* suggesting that they have effective mechanisms of dissipating excess light on rapid times scales (Veal et al., 2010). Additionally, *Symbiodinium* efficiently repairs the daily damage that occurs from photosynthesis (Gorbunov et al., 2001; Hoogenboom et al., 2006). Akin to other photosynthetic organisms, corals and their symbionts adapt to high and low light environments and have specific photosynthetic characteristics. The coral–algal symbiosis exhibit classic photosynthetic low and high light adaptation patterns: the coral–algal symbiosis under low light maximizes the amount of light processed through increased light-absorbing pigments and photosynthetic efficiencies to obtain high rates of photosynthesis under lower irradiances; in contrast, the coral–algal symbiosis under high light minimizes the amount of light processed through reduced pigments and photosynthetic efficiencies but higher maximum rates of photosynthesis under high irradiances (Falkowski and Dubinsky, 1981; Anthony and Hoegh-Guldberg, 2003a,b). Light is very dynamic and *Symbiodinium*, like all photosynthetic organisms, exploit a variety of photophysiological processes over a range of timescales to efficiently absorb and utilize light and prevent photoinhibition (**Table 1**).

### PHYLOTYPE

Because of the lack of morphological characteristics, it was originally believed that there was only one pandemic species of *Symbiodinium*, *S. microadriaticum* (Freudenthal, 1962). Upon greater consideration of physiology, biochemistry, ultra-structure, and other aspects, and more recently with molecular biology and phylogenetics, it has become apparent that *Symbiodinium* actually represents several divergent lineages known as clades A thru I (Stat et al., 2012). In addition to the symbiosis with corals, *Symbiodinium* are commonly found in symbiosis with other cnidarians (e.g., sea anemones) as well as Platyhelminthes, Mollusca, Porifera, and Foramniferans and even free-living (Stat et al., 2006).

Individual corals can host multiple phylotypes of *Symbiodinium* at the same time and through time. Recent techniques have shown that corals host 6–8 times greater diversity of *Symbiodinium* than previously assumed (Apprill and Gates, 2007) and can identify low abundance *Symbiodinium* (Mieog et al., 2007). The same species of coral found at different depths can harbor the same or different phylotypes of *Symbiodinium* (Iglesias-Prieto et al., 2004; Warner et al., 2006). Surprisingly, only one out of eight species of corals investigated showed a correlation between distinct coral microhabitat patterns and *Symbiodinium* phylotypes (van Oppen et al., 2001). Throughout the year, *Symbiodinium* phylotype varies both between clades and the proportion of different subclades (Suwa et al., 2008; Ulstrup et al., 2008). The diverse and variable assemblage of *Symbiodinium* within corals sets the stage for the inherent physiological capacity for photosynthesis and its responses to environmental changes.

### ABUNDANCE

The abundance of *Symbiodinium* is important because it may directly affect the amount of oxygen produced within corals cells and therefore the potential for ROS production. The irradiance regulates the density of *Symbiodinium* in corals, but *Symbiodinium* abundance also alters the light field within corals. Scleractinian corals typically host between 1 and 2 *Symbiodinium* cells per endoderm cell (Muscatine et al., 1998). Symbiont densities generally range from 1 to 4 × 10^6^ cells cm^-2^, but can be found as dense as 8 × 10^6^ cells cm^-2^ (Fagoonee et al., 1999; Fitt et al., 2000; Apprill et al., 2007). It is thought that the coral controls *Symbiodinium* density and its pigments through nitrogen limitation (Falkowski et al., 1993), although the mechanisms are not well understood (Davy et al., 2012). For a thorough discussion of *Symbiodinium* acquisition, regulation, expulsion, and degradation see the recent review by Davy et al. (2012). In laboratory experiments, *Symbiodinium* density can acclimate to new light intensities within 15 days (Roth et al., 2010). On coral reefs, *Symbiodinium* density changes inversely with seasonal light levels, decreasing in the summer and increasing in the winter and fall (Fagoonee et al., 1999; Fitt et al., 2000; Ulstrup et al., 2008), likely to optimize photosynthesis. During temperature stress, higher densities of *Symbiodinium* have been implicated in increasing the susceptibility of corals to bleaching because of the higher ROS production relative to corals’ antioxidant capacity (Cunning and Baker, 2013); however, high densities of *Symbiodinium* also result in significant self-shading, lower rates of oxygen evolution, and ultimately reduced ROS production. Because *Symbiodinium* absorbs light, irradiance declines the fastest where the layer of *Symbiodinium* are located within the coral tissue (Wangpraseurt et al., 2012). Thus, changes in *Symbiodinium* density, and in particular during bleaching, exacerbate the environmental stress on the remaining symbionts. Further research on populations of *Symbiodinium* including abundance, phylotype, and their physiological differences will elucidate the outcomes of the coral–algal symbiosis during environmental stress.

### ANTIOXIDANTS

Antioxidants neutralize ROS and play an important photoprotective role. Like corals and other photosynthetic organisms, *Symbiodinium* synthesize a variety of enzymatic antioxidants such as SOD, CAT, and ascorbate peroxidase (ASPX; Lesser and Shick, 1989; Shick et al., 1995). *Symbiodinium* in corals collected from high irradiance habitats have higher SOD, CAT, and ASPX activities than those collected from low irradiance habitats at the same depth (Lesser and Shick, 1989). Additionally, *Symbiodinium* in corals collected over a depth gradient show a decline in the activities of SOD, CAT, and ASPX with increasing depth, which may be related to the decrease in potential for oxidative stress (Shick et al., 1995). Similar to their hosts, activities of SOD and CAT in *Symbiodinium* increase with blue light and show a positive correlation with the diurnal cycle (Levy et al., 2006a,b). In culture, different phylotypes show distinct constitutive activities of SOD produced despite being grown under the same conditions (Lesser, 2011). Phylotypes with higher capacity for photoacclimation and thermal tolerance also have higher concentrations of the nonenzymatic antioxidant glutathione and xanthophylls (see Section “Carotenoids”; Krämer et al., 2011). MAAs have antioxidant activity in addition to absorbing UVR (**Table 2**). *Symbiodinium* synthesizes at least four MAAs in culture, but most MAAs are primarily passed to the host to be used as a first line of defense absorbing UVR before it can reach *Symbiodinium* (**Figure 2C**; Shick and Dunlap, 2002). For more details on antioxidants see the Section “Photobiology of Corals.”

### PHOTOSYNTHETIC PIGMENTS

Photosynthetic dinoflagellates including *Symbiodinium* have plastids derived from red algae. The primary photosynthetic pigments in *Symbiodinium* are chlorophyll *a*, chlorophyll *c_2_*, and peridinin (**Table 2**). While the core photosynthetic machinery is highly conserved among photosynthetic eukaryotes, the light-capturing pigments are diverse to match the particular light environment of the organism. *Symbiodinium* has two types of light-harvesting complexes: (1) the thylakoid membrane-bound chlorophyll *a*–chlorophyll *c_2_*–peridinin-protein-complex (acpPC) and (2) the water-soluble peridinin–chlorophyll *a* protein (PCP; Iglesias-Prieto et al., 1991, 1993). The chlorophylls primarily absorb high-energy blue light (∼430–460 nm), but chlorophyll *a* also absorbs red light (∼680 nm; **Table 2**; Bricaud et al., 2004). Peridinin expands the range of photosynthetically usable light of *Symbiodinium* because it has maximum absorption of blue-green light (∼480–500 nm) and a broad absorption spectra (∼450–550 nm; **Table 2**; Bricaud et al., 2004; Johnsen et al., 2011).

The majority of photosynthetic pigments are involved in absorbing and transferring light to the reaction centers of PSI and PSII. Photoacclimation processes can also involve changing the stoichiometry between antenna proteins and reaction centers and between photosystems. Within 15 days, *Symbiodinium* in corals photoacclimate by modifying the amount per cell of chlorophyll *a*, chlorophyll *c_2_*, and peridinin yet maintaining the same ratios of pigments (Roth et al., 2010). In culture, *Symbiodinium* also change the concentration of chlorophyll *a*, chlorophyll *c_2_*, and peridinin, but additionally change the ratios of photosynthetic pigments under different light conditions (Iglesias-Prieto and Trench, 1994; Robison and Warner, 2006; Hennige et al., 2009). This discrepancy between *Symbiodinium* in culture and symbiosis may suggest that the host modulates the light environment of *Symbiodinium* in symbiosis. Moreover, *Symbiodinium* can photoacclimate by changing the size of the photosynthetic unit by adjusting the abundances of PSI, PSII, acpPC, and PCP and the antenna size for each photosystem (Titlyanov et al., 1980; Falkowski and Dubinsky, 1981; Iglesias-Prieto and Trench, 1997; Hennige et al., 2009). A study of eight phylotypes of cultured *Symbiodinium* under two irradiance growth conditions suggests that the photoacclimation generally occurs by modifying the reaction center content rather than the effective antennae-absorption (Hennige et al., 2009). In shallow corals on reefs, chlorophyll *a* per cell decreases in the summer and increases in the winter (Fitt et al., 2000) and the ratio of chlorophyll *a* to chlorophyll *c_2_* can vary on a seasonal basis (Warner et al., 2002). Coral bleaching is defined as either a decrease in *Symbiodinium* density and/or a reduction in photosynthetic pigments (Coles and Jokiel, 1978; Warner et al., 1996; Hoegh-Guldberg, 1999; Roth et al., 2012), which alters the light scattering and absorption characteristics. Furthermore, there is a complex relationship between the increase in pigments and the decrease in optical absorption cross-section (the relationship between the rate of excitation delivered and the photochemical reaction) due to self-shading within the cell called the “package effect” (Kirk, 2010). The amount of packaging can vary between different phylotypes as well as under low and high light conditions (Hennige et al., 2009). Detailed studies on the changes in chlorophyll content of *Symbiodinium* under various light regimes for a variety of phylotypes will elucidate the packaging dynamics. In the coral–algal symbiosis, *Symbiodinium* pigment packaging is compounded by packaging of *Symbiodinium* within coral cells. The packing of pigments and cells adds complexity to the relationship between light absorption and pigments.

### CAROTENOIDS

Carotenoids are accessory pigments (tetraterpenoids) synthesized by photosynthetic organisms. There are two types of carotenoids: carotenes (pure hydrocarbons) and xanthophylls (hydrocarbons with oxygen).* Symbiodinium* synthesizes β-carotene and xanthophylls peridinin, diadinoxanthin, and diatoxanthin (**Table 2**). Carotenoids have a variety of roles including as accessory light-harvesting pigments, structural components of the light-harvesting complexes, antioxidants, and sinks for excess energy. Within minutes of high light, the xanthophyll cycle converts diadinoxanthin to diatoxanthin through de-epoxidation and the cycle is reversed in limiting light (Brown et al., 1999). Increases in xanthophyll de-epoxidation state, the ratio of diatoxanthin to the total xanthophyll cycle pool, are associated with photoprotection of the photosynthetic apparatus (Brown et al., 1999). *Symbiodinium* can increase the capacity for photoprotection by increasing the amount of β-carotene and xanthophylls relative to chlorophyll *a* (and vice versa); the increase occurs within 15 days during photoacclimation and within 5 days under temperature stress (Roth et al., 2010, 2012). Likewise, *Symbiodinium* in culture adjust the relative abundances of photoprotective pigments under different light environments (Hennige et al., 2009). Carotenoids provide important photoprotection for photosynthetic organisms under multiple timescales.

### PHOTOSYNTHESIS

Given the central role of photosynthesis in the coral–algal symbiosis, it is important to characterize a variety of photosynthetic related parameters. Quantifying photosynthesis under different light fields, generally referred to as photosynthesis to irradiance (P/E) curves, describes the dynamics of photosynthesis. From these data, the light compensation point (where photosynthesis and respiration are equal), photosynthetic efficiency (the slope under light-limiting conditions), saturating irradiance, and the photosynthetic maximum can be determined (see diagram in Osinga et al., 2012). Photoacclimation of eight phylotypes of cultured *Symbiodinium* under two growth irradiances provide evidence for highly variable bio-physical and bio-optical measurements (Hennige et al., 2009).* Symbiodinium* in culture photoacclimate by changing their maximum rate of net photosynthesis (*P*_max_), respiration rate and saturating irradiance (Iglesias-Prieto and Trench, 1994). In contrast, *Symbiodinium* in corals photoacclimate to new growth conditions primarily by changing saturating irradiances rather than changes in *P*_max_ (Anthony and Hoegh-Guldberg, 2003a). There are considerable differences in high and low light adapted corals including in *P*_max_, photosynthetic efficiency, saturating irradiance, respiration, and thylakoid packing (Falkowski and Dubinsky, 1981; Dubinsky et al., 1984; Anthony and Hoegh-Guldberg, 2003b). Changes in photosynthetic function are one of the first indicators of temperature stress of the coral–algal symbiosis (Iglesias-Prieto et al., 1992; Warner et al., 1996, 1999; Lesser, 1997; Lesser and Farrell, 2004; Roth et al., 2012).

Two of the most informative measurements in photobiology of the coral–algal symbiosis are the maximum quantum yield of photosynthesis (Φ) and its inverse the minimum quantum requirement (1/Φ). These measurements are calculated as the fraction of photosynthetically usable light absorbed by photosynthetic pigments used to drive photosynthetic activity (e.g., O_2_ evolved or CO_2_ assimilated). The theoretical limit of the minimum quantum requirement for photosynthetic organisms is eight photons absorbed per molecule of oxygen evolved (Wyman et al., 1987). Measuring the light absorbed by *Symbiodinium* in corals is challenging and at one point was regarded as impossible (Falkowski et al., 1990). Early measurements of Φ underestimated the absorption cross-section of chlorophyll because it was measured from freshly isolated *Symbiodinium* (Dubinsky et al., 1984; Wyman et al., 1987; Lesser et al., 2000) rather than in intact corals where the absorption is two to fivefold higher because of light scattering by the skeleton (Enríquez et al., 2005). Recent studies suggest that corals are efficient energy collectors and that the energy can be utilized close to the theoretical maximum (Rodríguez-Román et al., 2006; Brodersen et al., 2014). Φ varies within the coral (depth within the tissue), in corals collected from distinct light environments (high light vs. shade adapted) and in corals with different degrees of bleaching (Dubinsky et al., 1984; Rodríguez-Román et al., 2006; Brodersen et al., 2014). Additionally, the Φ is affected by the irradiance during measurement (Brodersen et al., 2014). Corals species and environmental history influence skeletal morphology, tissue thickness, and ultimately light scattering, which add to the variability in coral–algal photobiology. While this direct assessment of the efficiency of light utilization is an important measurement, it remains logistically cumbersome.

Chlorophyll fluorescence can be used as a proxy for many photosynthetic measurements and consequently the results can be interpreted as an indicator of coral health. Chlorophyll *a* fluorescence provides an understanding of the photochemical activity of PSII, photodamage, and photoprotection over temporal and spatial scales in a noninvasive manner (Warner et al., 2010). This review will briefly discuss some of the most widely measured fluorescence parameters of photosynthesis in the coral–algal symbiosis, but there are many types of fluorescence measurements that involve a variety of fluorometers that operate on different basic principles (reviewed in Cosgrove and Borowitzka, 2010; Warner et al., 2010). The maximum photochemical efficiency (quantum yield) of PSII (*F*_v_/*F*_m_) is measured in dark-acclimated corals and represents the maximum capacity of PSII. The effective or steady state photochemical efficiency of PSII (Δ*F*/*F*_m_^′^, Δ*F*^′^/*F*_m_^′^ or Φ_PSII_) is measured in the light-adapted state. Corals show a daily midday reversible decrease in Δ*F*/*F*_m_^′^ and *F*_v_/*F*_m_ associated with shunting energy away from photochemical reactions and into other pathways to prevent damage (**Figure 3**; Brown et al., 1999; Gorbunov et al., 2001). The functional absorption cross-section for PSII shows a diurnal pattern with a decline associated with peak irradiances during midday, which correlates with the decrease in Δ*F*/*F*_m_^′^, the increase in NPQ (see Section “Non-photochemical Quenching”) and the highest rate of net photosynthesis (Levy et al., 2006a). To maintain high rates of productivity under normal conditions, a percentage of PSII reaction centers (D1 protein) will become damaged during the day when the rate of damage exceeds the rate of repair, but PSII will be able to repair itself when the rate of repair exceeds the rate of damage in low light (nighttime; Gorbunov et al., 2001). *Symbiodinium* in corals photoacclimate by changing photosynthetic efficiency to new conditions within days in laboratory experiments (Roth et al., 2010), and over seasons on reefs (Warner et al., 2002; Ulstrup et al., 2008). Additionally, distinct microhabitats of the coral such as tops versus sides can show different photosynthetic efficiencies (Warner and Berry-Lowe, 2006). When *F*_v_/*F*_m_ declines over time, it implies that the rate of damage of PSII exceeds the rate of repair and damage has accumulated, which can lead to coral bleaching (Roth et al., 2012). The excitation pressure over PSII can be calculated as *Q*_m_ = 1 – [(Δ*F*/*F*_m_^′^ at peak sunlight)/(*F*_v_/*F*_m_ at dawn)] (Iglesias-Prieto et al., 2004). A low *Q*_m_ would signify a high proportion of PSII reaction centers are open and possible light limitation, whereas a high *Q*_m_ would signify that most PSII reaction centers are closed and there could be photoinhibition. A recent study showed that during a heat stress experiment, corals began bleaching when *Q*_m_ reached ∼0.4 and continued heat stress intensified the bleaching until the *Q*_m_ reached ∼0.8 (when measurements were no longer possible due to the low level of symbionts) while control corals maintained *Q*_m_ < 0.2 (Roth et al., 2012). Measuring chlorophyll fluorescence under various light regimes can also provide estimates of the relative electron transport rate (rETR) similar to P/E curves, but there are many problems and pitfalls with this approach (Warner et al., 2010; Osinga et al., 2012). Despite its limitations, measuring chlorophyll fluorescence is an important noninvasive methodology to assess the physiological state of *Symbiodinium* and thus the coral. For more information on the methodologies and the instrumentation mentioned in this section see recent reviews (Warner et al., 2010; Osinga et al., 2012).

### NON-PHOTOCHEMICAL QUENCHING

Excess energy harmlessly dissipated as heat, also called NPQ, is an important photoprotective mechanism. In **Figure 3**, the secondary outflow of the funnel is representative of NPQ pathways. NPQ includes all processes that decrease chlorophyll fluorescence yield apart from photochemistry and consists of energy-dependent quenching (qE), state transition quenching (qT), and photoinhibitory quenching (qI; Müller et al., 2001). NPQ processes are characterized according to their relaxation kinetics (Müller et al., 2001). In *Symbiodinium* in corals, >80% of excitation energy can be dissipated through NPQ (Gorbunov et al., 2001; Brodersen et al., 2014). Most of the energy is likely to be dissipated through qE rather than qT or qI (Niyogi, 1999).

#### Energy-dependent quenching

Turning on and off within minutes, qE is essential for coping with rapid changes in incident sunlight. In most eukaryotic algae, qE depends on a buildup of a transient ΔpH across the thylakoid membrane, a particular light-harvesting complex protein called LHCSR, and specific carotenoids of the xanthophyll cycle (Niyogi and Truong, 2013). However, LHCSR is not found in the Expressed Sequence Tag (EST) library of *Symbiodinium* (Boldt et al., 2012), which may suggest another mechanism for how qE is achieved in *Symbiodinium*.

#### State transition quenching

State transition quenching is the quenching that results from uncoupling the light-harvesting complexes from PSII to decrease the amount of light absorbed and transferred to the PSII reaction center in green algae and plants (Müller et al., 2001). In *Symbiodinium* under excess light, both light-harvesting complexes acpPC and PCP may dissociate from PSII to minimize PSII overexcitation (Hill et al., 2012). It is thought that the redistribution of acpPC from PSII to PSI could prevent photo-oxidative damage (and ultimately bleaching) in more tolerant phylotypes of *Symbiodinium* (Reynolds et al., 2008; Hill et al., 2012). State transitions are triggered by reversible phosphorylation of light-harvesting proteins and can occur in minutes and relax in tens of minutes (Müller et al., 2001; Eberhard et al., 2008). However, some studies on freshly isolated and cultured *Symbiodinium* have not observed the enhanced energy transfer to PSI (Warner et al., 2010). The relative role and specific mechanisms of qT in *Symbiodinium* as a photoprotection mechanism remain unknown.

#### Photoinhibitory quenching

Photoinhibitory quenching is the NPQ mechanism with the slowest relaxation kinetics and is poorly understood even in plants and green algae (Müller et al., 2001). During prolonged light stress, slowly reversible quenching occurs that is thought to result from both photoprotection and photodamage. qI relaxation generally occurs within hours in photosynthetic eukaryotes (Müller et al., 2001). More research is needed on the mechanisms of qI in *Symbiodinium* to fully understand the photoprotective pathways.

*Symbiodinium* utilizes a variety of processes on multiple timescales to protect its primary role of absorbing and processing light through photochemistry while avoiding oxidative stress. While much is understood about these mechanisms on a cellular and biochemical level, there is much to learn about how the various components and proteins are synthesized, regulated, assembled, and degraded. A recent study on gene expression in *Symbiodinium* (microarray containing 853 features) showed that 30% of genes show diurnal oscillations (Sorek et al., 2014). While some of these genes are associated with photosynthesis such as the peridinin-chlorophyll *a*-binding protein, many of the genes are uncharacterized (Sorek et al., 2014). Recent advances such as the *Symbiodinium* draft genome (Shoguchi et al., 2013) and transcriptome (Baumgarten et al., 2013) will permit new investigations into gene expression and posttranscriptional regulatory processes and should be paired with biochemical and physiological work to elucidate process on molecular, cellular, and biological levels.

## BEYOND LIGHT, INFLUENTIAL FACTORS IN PHOTOSYNTHETIC SYMBIOSES IN CORALS

Thus far, this review has focused on the effects of light on photosynthesis and the coral–algal symbiosis. Under specific conditions such as excess light, which is typical of sunny days in the shallow environment of reef-building corals (Gorbunov et al., 2001), there are additional abiotic and biotic factors that influence photosynthesis. Because all reef-building corals rely on energy from their symbionts (Osinga et al., 2011), the factors modifying photosynthesis are central to the health of the coral–algal symbiosis.

### ABIOTIC FACTORS

Abiotic factors that influence photosynthesis in the coral–algal symbiosis include availability of inorganic nutrients (in particular carbon), oxygen concentration, pH, and temperature, which are all modulated by water flow. Because corals are sessile, water flow dictates the rate of diffusion of gas exchange between the coral and the surrounding water by changing the thickness of the diffusive boundary layer. A coral extending its polyp may also affect the boundary layer, but those effects are uncharacterized. Abundance of dissolved inorganic carbon can the determine rates of photosynthesis and calcification in the coral–algal symbiosis (Falkowski et al., 1993; Marubini et al., 2003). Increased water flow has been shown to decrease the amount of oxygen within coral cells, which in turn increased the ratio of carboxylation to oxygenation catalyzed by Rubisco, and resulted in an augmentation of photosynthetic rate (Mass et al., 2010a). High flow and high irradiance result in faster growth rates of corals (Schutter et al., 2011). The combination of feeding corals (providing carbon, nitrogen, and phosphorus) and higher irradiance has an additive effect on coral growth (Osinga et al., 2011). Doubling carbon dioxide concentration, for example in ocean acidification experiments, does not increase photosynthesis or calcification in corals (Anthony et al., 2008). Corals may be able to regulate their internal pH and buffer against moderate changes in external pH and carbonate chemistry (Venn et al., 2013). Additionally, *Symbiodinium* can increase coral intracellular cytosolic pH through photosynthesis (Laurent et al., 2013).

Temperature anomalies can have serious consequences on the coral–algal symbiosis and the effects have been extensively studied as well as covered in recent reviews (Weis, 2008; Lesser, 2011). Temperature affects the activity of various enzymes and reactions involved in photosynthesis and ultimately the repair of critical proteins (Somero, 1995; Huner et al., 1998; Warner et al., 1999; Takahashi et al., 2004; Nobel, 2005). During temperature stress, changes in the fluidity of the thylakoid membrane affect photosynthetic electron transport capacity and dismantle the photosynthetic system resulting in a decomposition of the thylakoid structure (Iglesias-Prieto et al., 1992; Tchernov et al., 2004; Downs et al., 2013); as a result, *Symbiodinium* produces a high abundance of ROS, which is passed to the host (Weis, 2008; Lesser, 2011). Once the threshold of ROS that the coral can neutralize is exceeded, a cascade of events is triggered that results in coral bleaching (Weis, 2008; Lesser, 2011). Catastrophic coral bleaching often occurs during small increases in temperature over prolonged periods of time and frequently concurrent with calm, clear weather patterns (Baker et al., 2008; Weis, 2008; Lesser, 2011). Because intensity and duration of the temperature anomaly are important in coral bleaching, the National Oceanic and Atmospheric Administration (NOAA) Coral Reef Watch program monitors temperature via satellite to determine the cumulative stress on a particular area of coral reef using a thermal stress index called degree heating weeks (DHW; Strong et al., 2011). At a given location, the DHW represent the accumulation of how long an area has experienced higher than average temperatures, which are called HotSpots. For example, one week of a HotSpot of 1^∘^C is equivalent to one DHW. Significant bleaching occurs around four DHW, and widespread bleaching and mortality occurs around eight DHW (Strong et al., 2011). Because of the importance of light, the NOAA Coral Reef Watch program plans to integrate measurements of light, wind, water transparency, and waves among other parameters into the monitoring program (Strong et al., 2011).

### BIOTIC FACTORS

In addition to the influences of abiotic effects on photosynthesis, biotic effects possibly under host control can have important consequences on symbiotic photosynthesis but have not been extensively investigated. The most conspicuous distinction between *Symbiodinium* in symbiosis and in culture is the difference in morphology. *Symbiodinium* in symbiosis primarily are non-flagellate spherical cells (coccoid stage), while in culture they show diurnal morphological changes between the flagellate gymnodinioid stage (motile stage) in daylight and the coccoid stage at night (Muscatine et al., 1998; Yamashita et al., 2009). Additionally, *Symbiodinium* in culture, but not in symbiosis, make crystalline deposits of uric acid that align during the motile stage and are hypothesized to function as an eyespot (Yamashita et al., 2009).

In addition to these obvious differences that suggest that *Symbiodinium* in culture and in corals are in quite distinct states, there are also physiological and biochemical discrepancies. *Symbiodinium* in symbiosis has reduced metabolism as compared to *Symbiodinium* in culture, which was determined by comparing *Symbiodinium* freshly isolated from corals and those from cultures (Goiran et al., 1996). Additionally, the host may control photosynthetic rates and release of photosynthetic products. In freshly isolated *Symbiodinium* from corals, the amount of carbon fixed and released differed if the symbionts were in the presence or absence of synthetic “host” factors (free amino acids; Stat et al., 2008). Moreover, corals limit the growth rate of *Symbiodinium* in symbiosis; the doubling time of *Symbiodinium* in high light and low light corals is ∼70 and ∼100 days, respectively, which contrasts with a week in culture replete with nutrients (Falkowski et al., 1993). It is suspected that the corals control *Symbiodinium* growth through nitrogen limitation (Falkowski et al., 1993). Bacteria and cyanobacteria associated with corals may be able to provide both the host and the symbiont with nitrogen and affect the stability of the symbiosis (Lesser et al., 2007; Ceh et al., 2013). However, the effects of bacteria and viruses on *Symbiodinium* remain largely unexplored. The coral host may also be able to influence its symbiont on a biochemical level, which has been observed with *Symbiodinium* in sea anemones. In *Symbiodinium* from anemones, there are differences in photosynthetic proteins (e.g., Rubisco and peridinin–chlorophyll *a*-*c_2_*-binding protein) between cells in symbiosis versus in culture (Stochaj and Grossman, 1997). These studies provide evidence that research on *Symbiodinium* in culture may not reflect their behavior in symbiosis. While it is apparent that corals have some influence on *Symbiodinium* in symbiosis, the extent to which they regulate the activities of *Symbiodinium* and the mechanisms are unknown.

## DIVERSITY OF THE CORAL–ALGAL SYMBIOSIS

There is incredible genetic, biochemical, physiological, and ecological diversity within both scleractinian corals and *Symbiodinium* individually as well as within the symbiosis. Responses and tolerances to light and other environmental parameters by the host or its symbiont can vary based on phylotype as well as recent environmental and biological history (Ward et al., 2000; Robison and Warner, 2006; Warner et al., 2006; Middlebrook et al., 2008; Krämer et al., 2011). Due to the high diversity of reef-building corals, the exact number of species is unknown. However, hundreds of species of corals have been described based on morphology (Veron, 2000). There are considerable challenges in how to demarcate a species and it is likely that a combination of morphological and genetic (nuclear and mitochondrial markers) approaches will be necessary to understand the biodiversity of corals (Stat et al., 2012). The enormous morphological diversity of scleractinian corals also contributes to the varying degree of bleaching sensitivity (Veron, 2000; Marcelino et al., 2013). Vertical and lateral light gradients within corals can be different because of their unique tissue and skeletal characteristics (Wangpraseurt et al., 2012). Additionally, the internal light environment may be altered by different colors and abundances of FPs of different species (Salih et al., 2000; Alieva et al., 2008; Gruber et al., 2008; Roth et al., 2010). It is likely that there are many other cellular and biochemical distinctions between coral species, but they are largely underexplored. It is also difficult to tease apart physiological differences of corals alone because a healthy reef-building coral is one in symbiosis with *Symbiodinium*.

Like their hosts, *Symbiodinium* contains significant functional and genetic diversity. While it is known that there are nine clades of *Symbiodinium*, the number of species is unknown and there are a number of challenges in delineating species in this taxonomic group (Stat et al., 2012). In *Symbiodinium,* not only is there extensive intracladal diversity, but also substantial biochemical and physiological intercladal differences. *Symbiodinium* phylotype can determine photoacclimation and photosynthetic capacities as well as antioxidant activities (Savage et al., 2002; Robison and Warner, 2006; Hennige et al., 2009; Lesser, 2011). Additionally, phylotypes have different photoinhibition, photorepair mechanisms, and thylakoid lipid composition, which can determine thermal sensitivity (Tchernov et al., 2004; Ragni et al., 2010; Díaz-Almeyda et al., 2011; Krämer et al., 2011). A significant challenge is that the majority of *Symbiodinium* strains, particularly those most biologically relevant such as those that populate most of the corals from the Indo-Pacific, have not been able to be maintained in culture and thus not studied without their hosts.

There is an additional level of diversity in the coral–algal symbiosis because individual corals can host multiple types of *Symbiodinium* on various temporal and spatial scales. While it was originally believed that the symbiosis was mutualistic, it is now known that the coral-algal symbiosis spans the continuum from parasitism to mutualism (Lesser et al., 2013). Clade A and D are generally considered more parasitic while clade C is known as more mutualistic based on characteristics of carbon fixation and translocation (Stat et al., 2008; Cantin et al., 2009). Changes in environmental conditions and coral bleaching may create opportunities that favor specific or new symbioses. The significant diversity within each partner as well as in the symbiosis means that much of the diversity remains uncharacterized, but due to the biodiversity crisis this is an important area of research for understanding coral populations. Because the performance of the coral holobiont is dependent upon both partners of the coral–algal symbiosis, physiological and ecological studies would benefit from taxonomic identification of both partners.

## RECENT ADVANCES AND FUTURE DIRECTIONS

Recent advances in genomics, transcriptomics, translatomics, proteomics, lipidomics, and metabolomics (collectively referred to as the “omics”) will provide a fresh perspective into the coral–algal symbiosis and enhance the understanding of this complex relationship in a dynamic environment. The first coral genome was published in 2011 (Shinzato et al., 2011), which was followed by a draft of the larger genome of *Symbiodinium* (the anemone symbiont* S. minutum*) in 2013 (Shoguchi et al., 2013). Additionally, a number of scleractinian coral and *Symbiodinium* transcriptomes are available (e.g., Meyer et al., 2009; Bayer et al., 2012) and it is now possible to analyze both coral and symbiont transcriptomes simultaneously (Shinzato et al., 2014). For a full description of recent genomic and proteomic studies see the review by Meyer and Weis (2012). Quantitative gene expression studies under a variety of conditions will be important in establishing the key molecular players responsible for a range of processes and in particular for responses to light. For example, a recent global transcriptome investigation of corals in low pH conditions revealed that in addition to upregulation of calcification genes, genes for autotrophy and heterotrophy are upregulated (Vidal-Dupiol et al., 2013). Because “omics” studies encompass the collective characterization of an organism, they can provide new directions of focus that may have been overlooked or not considered.

“Omics” studies in *Symbiodinium* lag behind those on corals because of the size of the *Symbiodinium* genome (∼1500 Mbp; Shoguchi et al., 2013) and transcriptome (∼59,000 genes; Baumgarten et al., 2013). In addition to the large amount of cellular DNA they contain, there are a number of well-known genetic peculiarities to dinoflagellates such as having permanently condensed chromosomes, few or no nucleosomes and reduced plastid genomes (Hackett et al., 2004; Leggat et al., 2011b). A real-time PCR study of *Symbiodinium* showed no effect of diurnal changes in light levels or transfer from low to high light, on transcript abundance of reaction center proteins of both PSI and PSII, suggesting that posttranscriptional processes may be important for regulating proteins (McGinley et al., 2013). Most previous studies have focused on a small number of genes (e.g., Leggat et al., 2011a; McGinley et al., 2013; Sorek et al., 2013), but the tools are now available for quantitative transcriptome-wide studies. A recent study using RNA-seq on thermotolerant and sensitive phylotypes of *Symbiodinium* in the same coral host showed no detectable change in gene expression after a short heat stress despite evidence of symbiosis breakdown (Barshis et al., 2014). Another study on *S. microadriaticum* suggests that there is a low number of transcription factors, but that small RNAs (smRNAs) may be important for posttranscriptional regulation (Baumgarten et al., 2013). However, minimal changes were also observed in the endosymbiont enriched proteome from corals during temperature stress (Weston et al., 2012). This study found that 11% of peptides increased expression but that neither antioxidants nor heat stress proteins significantly increased expression under heat stress (Weston et al., 2012). Unexpectedly, temperature stress did cause an extraordinary 114-fold increase in a viral replication protein, which may suggest that viruses may play an important role in bleaching and/or disease when corals are stressed (Weston et al., 2012). System-level studies integrating “omics” with physiology will elucidate the genes, proteins, and regulatory factors relevant for photoacclimation and light stress of both partners of the symbiosis. Because these studies are unbiased, they can reveal new areas of focus such as the effects of viruses on *Symbiodinium* physiology. These technologies are advancing quickly and are now available on single cells (Wang and Bodovitz, 2010), which could reveal the heterogeneity of the mixed *Symbiodinium* assemblage as well as the physiological diversity within different layers of coral tissues. A new method has recently been developed for conducting automated massively parallel RNA single-cell sequencing (MARS-seq) on multicellular tissues (Jaitin et al., 2014), which could be used to examine the distinct cells of the coral tissue (e.g., cells with *Symbiodinium* and without). These exciting new technologies will offer a new characterization of the physiology of the coral–algal symbiosis.

In addition to the development of the “omics,” advances in traditional methodologies and interest by those with expertise in complex techniques can provide insights into the coral–algal symbiosis. The light-harvesting characteristics of *Symbiodinium* are important area of concentration because of the central role of photosynthesis in the health of the coral–algal symbiosis. Due to the unique spectroscopic properties of *Symbiodinium* light-harvesting complexes, acpPC and PCP have gained the attention of scientists who study photosynthesis in model photosynthetic organisms and employ a variety of sophisticated techniques and methodologies, which can be applied to *Symbiodinium*. The X-ray crystallography structure of PCP was recently determined (Schulte et al., 2009) because it is the only system where bound carotenoids (peridinin) outnumber chlorophylls. However, the structure of the acpPC complex is still unknown. A recent study has shown that PCP is protected from potential photodamage because peridinin has an extremely fast triplet state, which can instantaneously deplete triplet chlorophyll to prevent forming singlet oxygen (Niedzwiedzki et al., 2013a). Femtosecond time-resolved transient absorption spectroscopy of acpPC shows that the accessory pigments (most carotenoids and chlorophyll *c_2_*) are very effective at absorbing light and passing it to chlorophyll *a*, but the photoprotection capacity of acpPC remains questionable (Niedzwiedzki et al., 2013b). Light-harvesting complexes play a role in preventing the overexcitation and dissipation of excess energy and thus further research in these systems may provide important insight into coral–algal photophysiology. The recent interest in *Symbiodinium* by photosynthesis scientists from model organisms will help elucidate the cellular and biochemical mechanisms in this unique photosynthetic symbiont.

Other techniques that have provided valuable insight in other fields, yet are sorely lacking in the coral–algal field, include genetic transformation and coral cell lines in culture. Although a methodology for genetic transformation was described in *Symbiodinium* 16 years ago (ten Lohuis and Miller, 1998), there has been no progress reported since the initial study. Additionally, gene knockout or knockdown in the coral–algal symbiosis could reveal roles of critical proteins involved in responses to light and environmental stress, among other processes. Development of methodologies for RNA interference (RNAi), which are used for gene knockdown, are currently underway in *Symbiodinium* (Weber and Medina, 2012). Furthermore, a simplified system of coral cells in culture, both with and without *Symbiodinium*, would be a great asset to obtain a better grasp of the symbiosis. Almost all studies to date of *Symbiodinium* in culture have included cultures that are not axenic and therefore may have included bacteria, fungus and/or protists. Recently, clonal, axenic lines of *Symbiodinium* have been obtained (Xiang et al., 2013), and will be instrumental in understanding the roles of bacteria and viruses on *Symbiodinium*. Combinations of physiological, biochemical, and genetic studies under normal conditions, acclimation, and stress will provide the most insight into the coral–algal symbiosis.

Tremendous progress has been made over the last 30 years in the knowledge of the coral–algal symbiosis, and the recent advances in tools and techniques integrated with traditional methodologies will provide new insights into the symbiosis. Given that ∼75% of the world’s coral reefs are now considered threatened (Burke et al., 2011), now is the time to act swiftly in a coordinated, collaborative effort to make even greater strides in understanding the coral–algal symbioses for the protection and conservation of coral reef ecosystems.

## Conflict of Interest Statement

The author declares that the research was conducted in the absence of any commercial or financial relationships that could be construed as a potential conflict of interest.
